# Evaluation of Risk Factors Related to Ovarian Involvement in Endometrial Cancer Patients: A Retrospective Study

**DOI:** 10.31661/gmj.v13i.3587

**Published:** 2024-11-03

**Authors:** Setareh Akhavan, Azam Alsadat Mousavi, Nasim Zarifi, Shahrzad Sheikhhasani, Narges Zamani, Elahe Rezayof

**Affiliations:** ^1^ Department of Obstetrics and Gynecology, Faculty of Medicine,Vali -Asr Reproductive Health Research Center, Tehran University of Medical Sciences, Tehran, Iran; ^2^ Department of Obstetrics and Gynecology, School of Medicine, Vali Asr Hospital, Tehran University of Medical Sciences, Tehran, Iran; ^3^ Vali-E-Asr Reproductive Health Research Center, Family Health Research Institute, Imam Khomeini Hospital Complex, Tehran University of Medical Sciences, Tehran, Iran

**Keywords:** Endometrial Cancer, Ovarian Metastasis, Risk Factors, Cancer, Case-control Study

## Abstract

Background: Endometrial cancer (EC) is a cancer that occurs in women. This study aimed to determine the risk factors of Ovarian involvement (OI) in patients with EC and define the risk of developing an associated malignancy during follow-up after EC treatment. Materials and Methods: This retrospective study (n=280) was conducted to determine the risk factors of OI following EC in female patients with a definitive diagnosis of uterine cancer admitted to Imam Khomeini Hospital in Tehran over the preceding 15 years, between 2010 and 2024. We evaluated the roles of risk factors for OI, such as age, histological subtype, tumor grade, myometrial infiltration, tumor diameter, cervical infiltration, lympho vascular space invasion (LVSI), and positive peritoneal cytology for malignancy (P.P.C). Results: The results showed that there was a relationship between the risk factors of myometer, LVSI, cervix, serous, tube, intra-abdominal, complaint, lymph node and omentum between the two groups with and without OI (P0.05). Based on Univariate and Multiple Analysis, the results showed that Omentum Involvement, P.P.C and Serous Carcinoma were related to OI. Also, based on multivariate logistic regression The results confirmed that the following predictors were correlated with OI: P.P.C (OR=8.54, 95% CI=1.52_47.88, P-value=0.015), omentum involvement (OR=10.82, 95% CI=2.21_52.82, P-value=0.03), and serous carcinoma (OR=4.41, 95% CI=1.44_13.53, P-value 0.009). Conclusion: Myometer, LVSI, cervix, serous, tube, intra-abdominal, complaint, lymph node and omentum factors were related to OI. Evaluation of risk factors plays an important role in the management of EC patients who are exposed to ovarian metastasis, therefore, the diagnosis of risk factors can be helpful in treatment strategies.

## Introduction

Endometrial cancer (EC) is a cancer that occurs in women. [1][2]. According to the statistics published in the past decades, the prevalence of EC in the world is increasing. Accordingly, in 2020, about 4.5% of women’s cancers are EC. Also, EC-related deaths in the world are reported to be 97,000 [3]. Despite the expansion of knowledge and progress in the treatment of patients, the death rate of patients is still high [4][5]. When the diagnosis of EC in patients is delayed and the disease is detected in advanced stages, the survival rate of patients decreases significantly. Metastasis is an important issue that health workers are facing today in connection with EC patients. During EC progression, malignant cells metastasize to various organs. One of the most important organs is actually the ovary. Ovarian involvement (OI) has been observed in many EC patients. Despite infertility in the patient, this conflict also reduces their survival [6][7][8][9][10].

Recently, it has been shown that there are many risk factors related to EC metastasis in patients. Each of these risk factors, based on their nature, has a different effect on EC metastasis in ovarian metastasis [11][12][13]. These risk factors are acquired and inherited. Some study conducted asses risk factors in EC, But the important point is that the risk factors are different in each region based on the conditions of the patients [14][15][16].

Understanding the risk factors associated with OI is clinically significant for preserving fertility and other complications and improving prognosis. This issue impacts treatment decisions, outcomes, prognosis, and patient management. Some studies have been conducted to investigate this matter. However, many risk factors related to OI have not been identified. The current research focuses on assessing the risk factors associated with OI in patients with EC.

## Materials and Methods

Study Design

This retrospective study was conducted to determine the risk factors of OI following EC in female patients with a definitive diagnosis of uterine cancer admitted to Imam Khomeini Hospital in Tehran over the preceding 15 years, between 2010 and 2024. The diagnosis of EC in patients was confirmed based on the results of tests and also based on pathology results. This was while the patients were checked under the supervision of the relevant specialist.

Inclusion and Exclusion Criteria

In this study, 280 female patients diagnosed with uterine cancer (including endometrial and non-endometrial adenocarcinoma) were included for analysis. Patient data were extracted from the hospital’s archival records. Patients with a history of pre-existing ovarian diseases, infertility, prior use of hormonal medications such as oral contraceptive pills (OCPs), or pelvic infections preceding uterine cancer diagnosis were excluded.

Data Collection

Through a retrospective review of hospital records, comprehensive patient data were collected encompassing demographic features (age, gravidity, parity) and lesion-specific details (pathological grading, uterine cancer subtypes, myometrial invasion, cervical, lymph node, non-endometrioid histology, and vascular lymphatic space invasion). Patients were stratified into two groups based on evidence from imaging reports and biopsy findings: those with OI involvement and those without. Subsequently, these two groups’ baseline parameters were compared to discern factors associated with OI development.

Ethical Considerations

Patients' private information remained utterly confidential. Their diagnostic and treatment paths were not disturbed, and no extra cost was imposed on the patient. Any exploitation of patients' information was done with their permission and the approval of the center's research assistant (IR.TUMS.IKHC.REC.1402.299).

Method for Calculating Sample Size

Based on the study by Ashrafganjoei et al. [17], which reported a prevalence of OI at 1.11%, and considering a confidence level of 95% and a precision level of 5%, the minimum required sample size for the study was determined to be equivalent to 152 cases.

N=P ˟ (1 - P) ˟ Z1-α/2 2/d2

P=0.111, α=0.05, Z1-α/2= 1.96, d=0.05

N=152

Statistical Analysis

The results were reported as mean ± standard deviation (mean ± SD) for quantitative variables and percentages for categorical variables. The data distribution was not normal, so the Mann-Whitney test was used. Mann-Whitney was applied to compare quantitative variables, while chi-square test was used for qualitative variables. Multiple logistic regression analysis was conducted to identify risk factors related to OI. A significance level of 0.05 or lower was considered statistically significant. Statistical analysis was carried out using SPSS version 23 software (IBM Corp., Armonk, NY., USA).

## Results

**Table:** Table1. Demographical Information of Patients

variables		Ovarian involvement		P-value
		Yes	No	
	Age (year)	57.78 11.53	57.09 9.91	0.88
	0-3	16(57.1)	122(47.5)	
Gravid	4-6	7(25)	87(33.9)	0.57
	>6	5(17.9)	48(18.7)	
	0-3	17(60.7)	136(52.9)	
Parity	4-6	8(28.6)	87(33.9)	0.73
	>6	3(10.7)	34(13.2)	
	Endometrioid	19(67.9)	234(91.1)	
Type	Serous	6(21.4)	19(7.4)	<0.001*
	Clear cell	1(3.6)	3(1.2)	
	Carcinoma sarcom	2(7.1)	1(0.4)	
	I	8(28.6)	116(45.1)	
Grade	II	5(17.9)	75(29.2)	0.008*
	III	15(53.6)	66(25.7)	

• As P<0.05

**Table:** Table2. Pathological Information of Patients

variables		Ovarian involvement		P-value
		Yes	No	
Myometer	>50	9(32.1)	145(56.9)	0.01*
	<50	19(67.9)	110(43.1)	
LVSI	Yes	20(71.4)	98(38.1)	0.001*
	No	8(28.6)	159(61.9)	
Cervix	Yes	16(57.1)	48(18.7)	<0.001*
	No	12(42.9)	209(81.3)	
Serous	Yes	14(50)	16(6.2)	<0.001*
	No	14(50)	241(93.8)	
Tube	Yes	13(46.4)	16(6.2)	<0.001*
	No	15(53.6)	241(93.8)	
Intra-abdominal	Yes	8(28.6)	8(3.1)	0.03*
	No	20(71.4)	249(96.9)	
	AUB	4(14.3)	46(17.9)	
	mass	1(3.6)	0(0)	
	monometro	3(10.7)	1(0.4)	
	none	0(0)	2(0.8)	
complaint	pain	2(7.1)	5(1.9)	<0.001*
	pain.edema	1(3.6)	0(0)	
	PMB	17(60.7)	202(78.6)	
	UIB	0(0)	1(0.4)	
Lymph node	Yes	7(25)	23(8.9)	0.009*
	No	21(75)	234(91.1)	
Omentum	Yes	4(14.3)	12(4.7)	0.03*

• As P<0.05

**Table:** Table3. Variables Associated with Ovarian Metastasis

**Variable**	**Univariate Analysis:** **Odds Ratio (95%CI)**	**Multiple Analysis:** **Odds Ratio (95%CI)**	**P-value**
Omentum Involvement	21.36 (5,50-81-34)	10.82 (2.21-52.82)	0.03*
Cervical stromal Involvement	2.14 (1.04-4.4)	0.84 (0.29-2.43)	0.75
P.P.C	10.96 (3.38-35.46)	8.54 (1.52-47.88)	0.015*
Full myometrial invasion	5.2 (1.51-17.99)	2.69 (0.44-16.47)	0.28
Serous Carcinoma	5.6 (2.36-13.6)	4.41 (1.44-13.53)	0.009*
Myometrial Involvement (>50%)	2.06 (1.04-3.09)	1.020 (0.33-3.17)	0.96
Myometrial Involvement (<50%)	0.3 (0.13-0.65)	0.49 (0.15-1.5)	0.23
Parametrial Involvement	2.7 (1.06-7.22)	0.46 (0.09-2.43)	0.36
Uterine serosa extension	5.6 (2.16-14.6)	1.46 (0.3-7.12)	0.63
Lymph node Involvement	2.71 (1.10-6.68)	0.82 (0.22-0.3)	0.77
Fallopian tube Involvement	6.14 (2.53-14.9)	1.73 (0.36-8.31)	0.48

• As P<0.05

In this study, the 280 data of female patients diagnosed with uterine cancer (including endometrial and other types of sarcoma) were extracted from the hospital's archival files and analyzed.

Demographical Information of Patients

Data analysis showed that there was no significant relationship between age, gravid and parity between the two groups (P>0.05). However, it was found that the difference in the frequency of grades I, II and III in the two groups was statistically significant (P=0.008). Also, in relation to the type of pathology, there were three types of endometrioid, serous, clear cell, and carcinoma sarcoma, and the difference in frequency between the two groups was statistically significant (P<0.001, Table-1).

Pathological Information of Patients

The results showed that the frequency of Myometer, LVSI, cervix, serous, tube, intra-abdominal, lymph node and omentum was different between the two groups, so these differences were statistical significant (P<0.05). Based on this, it was found that their frequency was higher in the group without OI. Also, in terms of complaints, which were divided into different categories, a comparison was made between the two groups, which was statistical statistically (P<0.001, Table-2).

Univariate and Multiple Analysis of Risk Factors

In univariable analysis, deep (>50%) myometrial invasion (OR=2.06, 95% CI: 1.04_3.9, P=0.96), non-deep (<50%) myometrial invasion (OR=0.3, 95% CI: 0.13_0.65, P=0.23), omentum involvement (OR=21.36, 95% CI: 5.50_82.94, P=0.03), P.P.C (OR=10.96, 95% CI=3.38_35.46, P=0.015), full myometrial invasion (OR=5.2, 95% CI=1.51_17.99, P=0.28), cervical stromal involvement (OR=2.14, 95% CI=1.04_4.40, P=0.75), parametrial invasion (OR=2.7, 95% CI=1.06_7.22, P=0.36), uterine serosa extension (OR=5.6, 95% CI=2.16_14.60, P=0.63), fallopian tube involvement (OR=6.14, 95% CI= 2.53_14.90, P=0.48), lymph node involvement (OR=2.71, 95% CI=1.10_6.68, P=0.77), and serous carcinoma (OR=5.6, 95% CI=2.36_13.60, P=0.006) showed significant differences (Table-3).

ROC Curve Analysis

**Figure-1:**
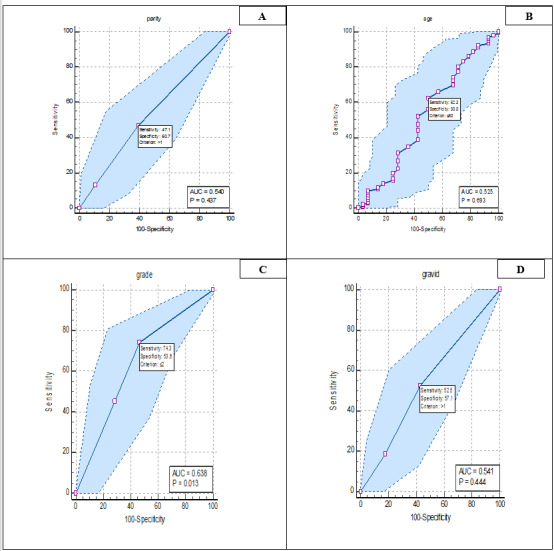
Figure 1. Roc curve analysis related demographic variables. A: parity, B: Age, C: grade and D: gravid.

In Figure-1, ROC curve analysis was analyzed for the diagnostic value of demographic variables in patients. Based on this, the results showed that only the variable and grade was significant (P=0.013). It was also determined the sensitivity and specificity for age (sensitivity: 62.26%, specificity: 50%), gravid (sensitivity: 81.32%, specificity: 57.14%), parity (sensitivity: 47.08%, specificity: 60.71%), and grade (sensitivity: 74.32%, Specificity: 53.57%) was calculated.

**Figure-2:**
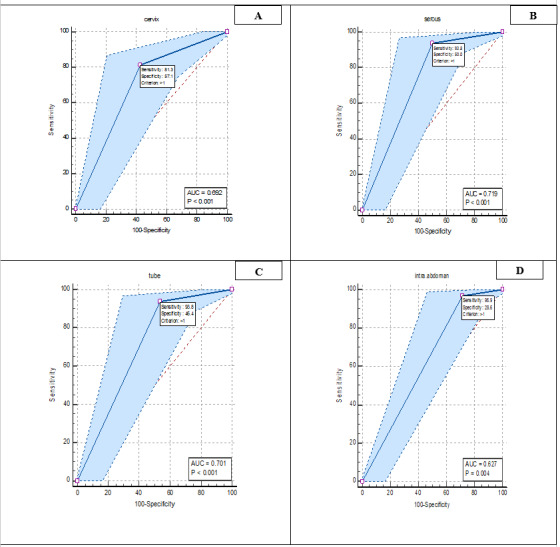
Figure 2. Roc curve analysis related pathological variables. A: cervix, B: serous, C: tube, and D: inta-abdominal.

In Figure-2, ROC curve analysis was analyzed for the diagnostic value of pathology variables in patients. The results showed that all four variables of cervix, serous, tube and intra-abdominal were significant (P<0.05). Also, sensitivity and specificity for cervix (sensitivity: 81.32%, specificity: 57.14%), serous (sensitivity: 93.77%, specificity: 50%), tube (sensitivity: 93.77%, specificity: 43.46%), and intra-abdominal (sensitivity: 96 .89%, Specificity: 28.57%) was calculated.

## Discussion

The main objective of present study was to identify the factors that cause OI in EC and mitigate this complication by addressing modifiable factors. Identifying these risk factors is critical to developing effective treatment strategies and improving patient management. The results showed that the frequency of Myometer, LVSI, cervix, serous, tube, intra-abdominal, lymph node and omentum was different between the two groups, so these differences were statistical significant (P<0.05).

Based on this, it was found that their frequency was higher in the group without OI. Also, in terms of complaints, which were divided into different categories, a comparison was made between the two groups, which was statistical statistically (P<0.001). In univariable analysis, deep (>50%) myometrial invasion, non-deep (<50%) myometrial invasion, omentum involvement, P.P.C, full myometrial invasion, cervical stromal involvement, parametrial invasion, uterine serosa extension, fallopian tube involvement, lymph node involvement, and serous carcinoma showed significant differences In addition, our study confirms that myometrial invasion is a significant factor associated with OI. Both deep (>50%) and non-deep (≤50%) myometrial invasion were significantly associated with outcome, with deep invasion representing a higher risk. This is consistent with previous clinical studies demonstrating an association between deep myometrial invasion and OI in patients with EC [18][19][20] [14][21][22],. High histologic grade (G2-G3) and lymphatic space invasion (LVSI) were also identified as valuable indicators for assessing OM, consistent with our findings and previous studies [23][24].

This finding is consistent with a 2022 study that identified age as a variable influencing OI in EC patients [25]. It is essential to consider age-related differences when developing treatment strategies for EC patients. In a similar study conducted by Matoba et al., they reported that in the analysis, pathological findings indicating that Lymphatic space invasion (LSI), cervical stromal involvement (CSI) peritoneal dissemination, and ovarian swelling (OvS) were cause progression of disease. Additionally, type 2 histologic type, LSI, CSI, and OvS were significantly more common in cases with OM in stage I and II without adnexal pathological factors. In multivariate analysis, LSI, CSI, and OvS emerged as significant risk factors for OI [23].

Type II EC cancer (e.g., serous or clear cell carcinoma) is more likely to metastasize to the ovaries compared to Type I (endometrioid) EC [23][26]. In a 2023 study of 1240 patients with EC, Qian Li and Xin Zhang concluded that deep myometrial invasion, lymph node metastasis, and higher CA125 levels may be independent high-risk factors for OI in patients with EC. OI has a higher influence on the prognosis in patients with low-risk EC [27]. OI in EC patients significantly impacts prognosis. It is associated with poorer outcomes and lower overall survival rates. The presence of OI often necessitates more aggressive treatment strategies and can affect decisions regarding fertility preservation [20][27][28].

One large multicenter retrospective study conducted by Ignatov et al. [20] in 2329 patients with EC, the 5-year overall survival rate was significantly lower in patients with OI (51.9%) than those without (84.6%). They found that OI in EC patients is associated with poorer prognosis and reduced survival rates compared to those without OI [20]. EC is still the most common cancer of the female reproductive system and is responsible for around 20% of deaths worldwide [29]. The high invasiveness and metastatic capacity of this cancer, especially to the ovaries, requires comprehensive treatment strategies, including primary hysterectomy, bilateral salpingo-oophorectomy, and pelvic and para-aortic lymphadenectomy.

This standard treatment protocol is critical even for young patients with low-risk, early-stage EC, where oophorectomy is performed as part of the surgery to reduce the risk of metastasis.

Despite the solid findings, this study faced some issues, including a limited number of eligible patients and a retrospective case-control design. Inconsistent pathology reports of precursor lesions such as endometrial hyperplasia or concurrent ovarian endometriosis could have influenced the final diagnosis. Therefore, future research should focus on developing novel genetic tools to accurately classify patients with complex symptoms and strive for a more accurate research database.

## Conclusion

Overall, this study underscores the significant risk factors for OI in EC, which include deep and non-deep myometrial invasion, omentum involvement, ascites, full stromal cervical involvement, parametrial invasion, uterine serosa extension, fallopian tube involvement, lymph node involvement, and serous carcinoma. P.P.C, omentum involvement, and serous carcinoma are powerful predictors.

Identifying these risk factors is crucial for developing effective treatment strategies, especially for preserving fertility in young women. Future research should focus on larger, prospective studies and the development of genetic tools to enhance diagnostic precision and treatment outcomes.

## Acknowledgement

We wish thank you of all our colleague in Isfahan university of medical science.

## Conflict of Interest

The authors declare that they have no conflict of interest.
